# The mitogenome of the Northern Hemisphere native terrestrial flatworm *Rhynchodemus sylvaticus* (Leidy, 1851) (Platyhelminthes, Geoplanidae)

**DOI:** 10.1080/23802359.2025.2515435

**Published:** 2025-06-08

**Authors:** Romain Gastineau, Christian Otis, Brian Boyle, Leigh Winsor, Jean-Lou Justine

**Affiliations:** a Institute of Marine and Environmental Sciences, University of Szczecin, Szczecin, Poland; b Plateforme d’Analyse Génomique, Institut de biologie intégrative et des systèmes, Université Laval, Québec City, Quebec, Canada; c College of Science and Engineering, James Cook University, Townsville, Queensland, Australia; d ISYEB, Institut de Systématique, Évolution, Biodiversité (UMR7205 CNRS, EPHE, MNHN, UPMC, Université des Antilles), Muséum National d’Histoire Naturelle, Paris, France

**Keywords:** Rhynchodemini, continenticola, premature ending, stop codon, phylogeny

## Abstract

*Rhynchodemus sylvaticus* (Leidy, 1851) is a tiny terrestrial flatworm of the subfamily Rhynchodeminae (Platyhelminthes, Geoplanidae) generally found in Europe and North America. Its mitogenome is 16,891 bp long, contains 12 protein-coding genes, two rRNA genes and 21 tRNA genes. No *tRNA-Thr* was found, *ND6* starts with ATT, *cob* is longer at its 3’ ending. Unlike the other eight species of Rhynchodeminae with sequenced mitogenomes, there is a real stop codon for *ND5.* Also, the position of tRNA-Met differs. Multiprotein phylogeny shows *R. sylvaticus* within a clade including species of the tribe Rhynchodemini from Oceania, but distinguished by a long branch.

## Introduction

Recently, the mitogenomes of several invasive terrestrial flatworms have been sequenced (Solà et al. 2015; Gastineau et al. 2019, 2020; Gastineau and Justine 2020; Justine et al. 2020, 2022, 2024; Soo et al. 2023; Gastineau, Lemieux, et al. 2024). These sequencing results showed that taxa belonging to the same subfamily might share common genomic characters, as exemplified by the subfamily Rhynchodeminae Graff, 1896. All species of Rhynchodeminae examined so far display a 32 bp overlap between *ND4L* and *ND4*, a central extension of the *cox2* gene and a premature stop of *ND5* because of the presence of a *tRNA-Ser* (Gastineau, Lemieux, et al. 2024), but all these sequences have been obtained from species that originate from the Southern Hemisphere. There are also species of Rhynchodeminae from Northern Hemisphere, such as the elusive species *Rhynchodemus sylvaticus* (Leidy, 1851) which is considered native to the USA (Hyman 1943) but also widely spread in Europe, although the conditions of its introduction to Europe are yet unclear. Occurrences reported in iNaturalist (https://www.inaturalist.org/observations?taxon_id=484654) suggest that the species has now been introduced into Australia, Belize, Brazil, China, the Cook Islands, Hawaii, Iceland, India, Japan, New Zealand, Puerto Rico, Thailand, Trinidad, and Tobago and Vietnam. The aim of the current article is to sequence the mitogenome of *R. sylvaticus* and use it to assess the phylogenetic position of this species within Rhynchodeminae.

## Material and methods

**Figure 1.**
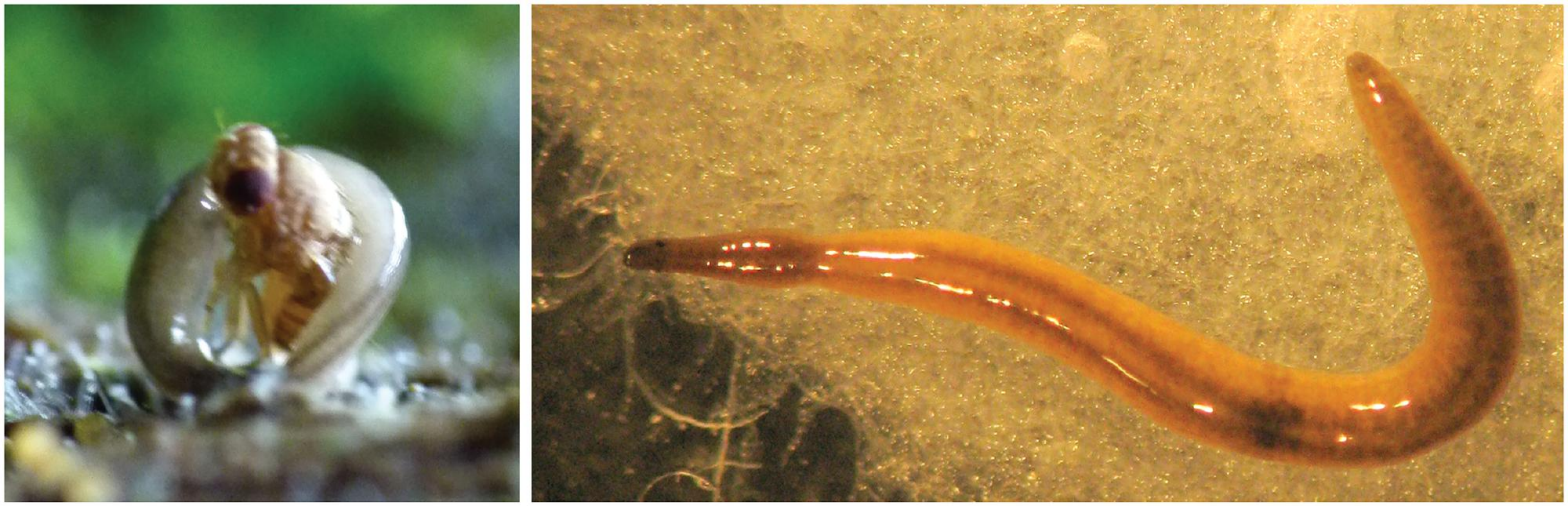
Live *Rhynchodemus sylvaticus* in a terrarium. Left: predation on a wingless *Drosophila hydei*. Right: dorsal view; the head is on the left, the two eyes are visible. Unscaled; total length of extended specimen ca. 10 mm. Photographs by Benjamin Loppin, reproduced with his permission.

*Rhynchodemus sylvaticus* was collected in 2014 in the vicinity of Lyon, France, by Benjamin Loppin, inside his home terrarium used for exotic frogs, which were fed with mutant apterous flies *Drosophila hydei* Sturtevant, 1921 (latitude 45.7293, longitude 4.825) (Figure 1). One specimen was sent to the Muséum National d’Histoire Naturelle, Paris (MNHN) and registered within the collections under the accession number MNHN JL195B (curator: Pr. Jean-Lou Justine, jean-lou.justine@mnhn.fr). *Rhynchodemus sylvaticus* is commonly considered a pest by exotic frog breeders or terrarium owners (Jaskuła et al. 2019; Anonymous 2024). Specimen was sent to the Genomic Analysis Platform of the Laval University, Québec, Canada. DNA was extracted using the CTAB-chloroform protocol as described in Gastineau et al. (2023). A total amount of 1.0 μg of DNA was retrieved. The distribution of the size of fragments in the DNA preparation was determined using a Femto Pulse from Agilent (Santa Clara, CA, USA). The library was produced with 500 ng of DNA, preliminarily broken with a Covaris M220 (Covaris, Woburn, MA, USA) and an NEBNext Ultra II DNA Library Prep Kit for Illumina from New England Biolabs (Ipswich, MA, USA). A total amount of ca 205 M 150 bp paired-end reads was obtained from the AVITI Sequencing System with a PE 150 sequencing kit Cloudbreak Freestyle High Output (Element Biosciences, San Diego, CA, USA). Raw reads were cleaned using fastp (Chen et al. 2018) with a size threshold of 125 bp, leaving a total of ca. 175 M paired-end reads. Assembly was done using SPAdes 4.0 (Bankevich et al. 2012) with a k-mer parameter of 125. The contig corresponding to the mitochondrial genome was extracted from the contigs file using standalone blastn queries (Camacho et al. 2009). Genes were annotated with the help of MITOS (Donath et al. 2019), except for the rRNA genes that were found by alignments with reference sequences from other species of Rhynchodeminae. ARWEN v1.2 was also used to verify the positions of tRNA (Laslett and Canbäck 2008). The map of the mitochondrial genome was obtained from the OGDRAW online portal (Lohse et al. 2013). Maximum likelihood analysis was conducted by appending recently published multiprotein datasets (Gastineau, Murchie, et al. 2024; Justine et al. 2024) with the sequences of the 12 mitochondrial proteins of *R. sylvaticus*. Each protein sequence was aligned independently using MAFFT 7 (Katoh and Standley 2013) with the -auto option. Alignments were trimmed with trimAl (Capella-Gutiérrez et al. 2009) and the -automated1 option. Sequences were then concatenated using Phyutility 2.7.1 (Smith and Dunn 2008). The best model of evolution was obtained on each concatenated alignment by using ModelTest-NG (Darriba et al. 2020) with default options and was MTZOA+I + G4 + F. Phylogeny was performed with IQ-TREE 2.2.0 (Minh et al. 2020) with 1000 ultrafast bootstrap replicates.

## Results

**Figure 2.**
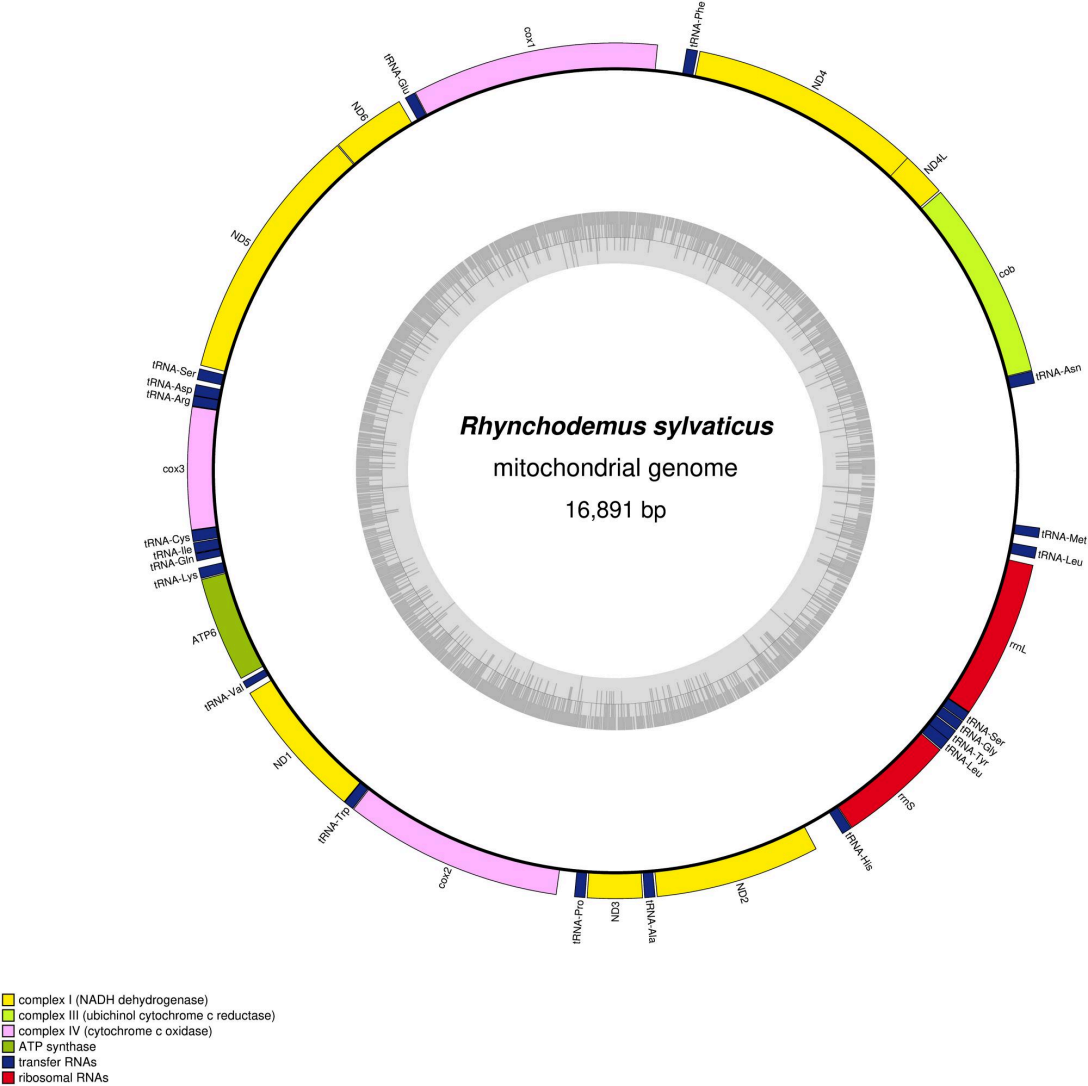
The mitogenome of *Rhynchodemus sylvaticus*. The mitogenome is 16,891 bp long and contains 12 protein-coding genes, two rRNA genes and 21 tRNA genes. The types of genes are represented by boxes of different colors (legend in caption). The grey circle represents the GC content. The mitogenome is represented as circular, although it could not be circularized after assembly.

**Figure 3.**
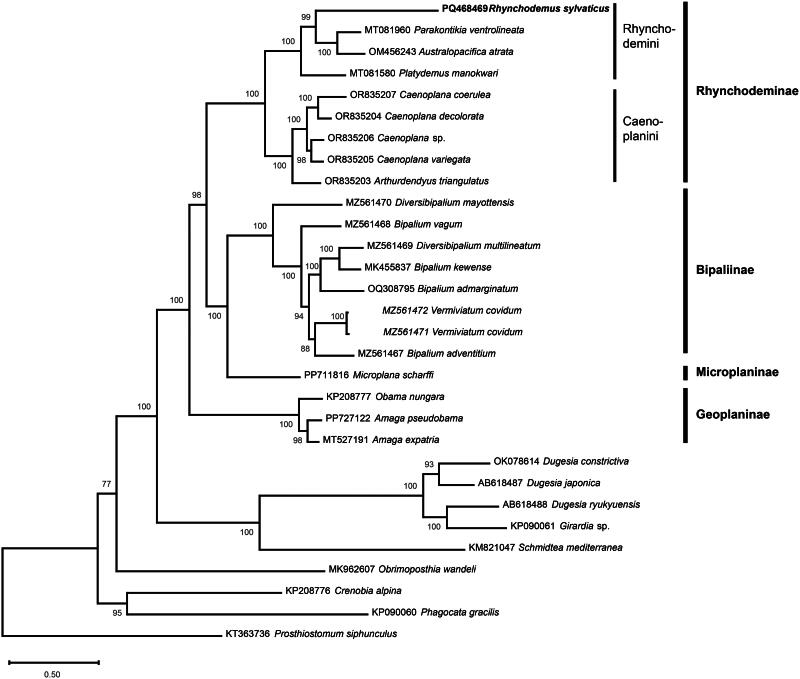
Maximum likelihood phylogenetic tree based on concatenated amino acid sequences of 12 mitochondrial proteins. Support values indicated at the nodes. The following sequences were used: *Prosthiostomum siphunculus* (KT363736) as an outgroup (Aguado et al. 2016), *Phagocata gracilis* (KP090060) (Ross et al. 2016), *Crenobia alpina* (KP208776) (Solà et al. 2015), *Obrimoposthia wandeli* (MK962607) (Yang et al. 2019), *Schmidtea mediterranea* (KM821047) (Ross et al. 2016), *Girardia* sp. (KP090061) (Ross et al. 2016), *Dugesia ryukyuensis* (AB618488) (Sakai and Sakaizumi 2012), *Dugesia japonica* (AB618487) (Sakai and Sakaizumi 2012), *Dugesia constrictiva* (OK078614) (Wang et al. 2022), *Amaga expatria* (MT527191) (Justine et al. 2020), *Amaga pseudobama* (PP727122) (Justine et al. 2024), *Obama nungara* (KP208777) (Solà et al. 2015), *Microplana scharffi* (PP711816) (Gastineau, Murchie, et al. 2024), *Bipalium adventitium* (MZ561467) (Justine et al. 2022), *Vermiviatum covidum* (MZ561471 and MZ561472) (Justine et al. 2022), *Bipalium admarginatum* (OQ308795) (Soo et al. 2023), *Bipalium kewense* (MK455837) (Gastineau et al. 2019), *Diversibipalium multilineatum* (MZ561469) (Justine et al. 2022), *Bipalium vagum* (MZ561468) (Justine et al. 2022), *Diversibipalium mayottensis* (MZ561470) (Justine et al. 2022), *Arthurdendyus triangulatus* (OR835203) (Gastineau, Lemieux, et al. 2024), *Caenoplana variegata* (OR835205) (Gastineau, Lemieux, et al. 2024), *Caenoplana* sp. (OR835206) (Gastineau, Lemieux, et al. 2024), *Caenoplana decolorata* (OR835204) (Gastineau, Lemieux, et al. 2024), *Caenoplana coerulea* (OR835207) (Gastineau, Lemieux, et al. 2024), *Platydemus manokwari* (MT081580) (Gastineau et al. 2020), *Australopacifica atrata* (OM456243) (Gastineau et al. 2022), *Parakontikia ventrolineata* (MT081960) (Gastineau and Justine 2020) and *Rhynchodemus sylvaticus* (PQ468469) (this study).

The mitogenome retrieved from the contigs file is 16,891 bp, contains 12 protein-coding, two rRNA and 21 tRNA genes (GenBank: PQ468469) (Figure 2). The coverage depth plot of the mitochondrial genome of *R. sylvaticus*, as obtained by plotCoverage, is available in Supplementary data. No *tRNA-Thr* could be found. The *cox2* gene is unusually long with a size of the putatively encoded protein of 466 amino acids (AA). *ND4L* overlaps *ND4* by 32 bp. No canonical start codon accepted for genetic code 9 (echinoderm and flatworm mitochondrial code) could be evidenced for *ATP6* and the gene seems to start with an ATT codon. The *cob* gene seems longer at its 3′ end. It was not possible to find any premature stop because of the presence of a tRNA. Alignment with reference sequences suggested that the commonly encountered TAA stop codon might have been altered into a GAA codon. The putative encoded Cob protein is 423 AA long, while it was found to range between 358 AA to 376 AA among other geoplanids. Conversely, a real canonical stop codon was found for *ND5*, with a protein size of 551 AA, which is similar to the size observed on the other species of Geoplanidae, regardless of the subfamily concerned. There is a *tRNA-Ser* located after *ND5*, but separated from its stop codon by 19 bp. Also, the position of *tRNA-Met* differs from other species of Rhynchodeminae, as it is located after *rrnL* instead of between *ND2* and *tRNA-His*. The position of *tRNA-Met* was also verified with ARWEN and by alignment of the mitogenome with the corresponding gene of *Parakontikia ventrolineata* (Dendy, 1892) (MT081960), returning the same result. In the phylogenetic tree (Figure 3), *R. sylvaticus* was found as sister species to a clade that contains *Pa. ventrolineata* and *Australopacifica atrata* (Steel, 1897), with maximum support, and this three-taxon clade was sister-group to *Platydemus manokwari* de Beauchamp, 1963. The branch of *R. sylvaticus* was very long, the longest among all geoplanids.

## Discussion

The two most noticeable results of our study are the phylogenetic position of *R. sylvaticus* among Rhynchodeminae and the structural differences of the mitogenome. The impact of the premature termination of *ND5* by *tRNA-Ser* is probably best exemplified by *A. atrata* (OM456243). The length of the *ND5* encoded protein for this species was found to be 550 AA with the premature termination, which is similar to *R. sylvaticus* for example. Without premature termination, the encoded protein would be 596 AA long and the gene would overlap half the sequence of a *tRNA-Asp*. It would be tempting to see the natural *ND5* stop codon shared by *R. sylvaticus*, Microplaninae, Bipaliinae and Geoplaninae as a primitive character that disappeared among other taxa of Rhynchodeminae. However, this idea is not supported by the phylogeny, which indicates instead a position of *R. sylvaticus* within the Rhynchodemini but with a very long branch. It should be noted that the phylogeny presented in Alvarez-Presas et al. (2014) based on concatenated *cox1* and nuclear *28S* returned similar results, also showing a marked distance from the common node. We also regard as being worthy of further investigations the difference of position of *tRNA-Met* and the extra-length of *cob.* These results advocate for additional efforts to study the genomics of Northern Hemisphere species of Rhynchodeminae.

## Supplementary Material

Supplemental Material

## Data Availability

The genome sequence data that support the findings of this study are openly available in GenBank of NCBI at [https://www.ncbi.nlm.nih.gov/nuccore/PQ468469] (https://www.ncbi.nlm.nih.gov/) under the accession no. PQ468469. The associated **BioProject**, **SRA**, and **Bio-Sample** numbers are PRJNA1172996, SRX26383929 and SAMN44286863, respectively.
