# Supplementary material for: The mitogenome of the Northern Hemisphere native terrestrial flatworm Rhynchodemus sylvaticus (Leidy, 1851) (Platyhelminthes, Geoplanidae)
Source: Mitochondrial DNA B Resour. 2025 Jun 8;10(7):558–62. doi: 10.1080/23802359.2025.2515435 (PMC12150643; doi:10.1080/23802359.2025.2515435)
Supplement: Supplemental Material [file TMDN_A_2515435_SM0220.docx]

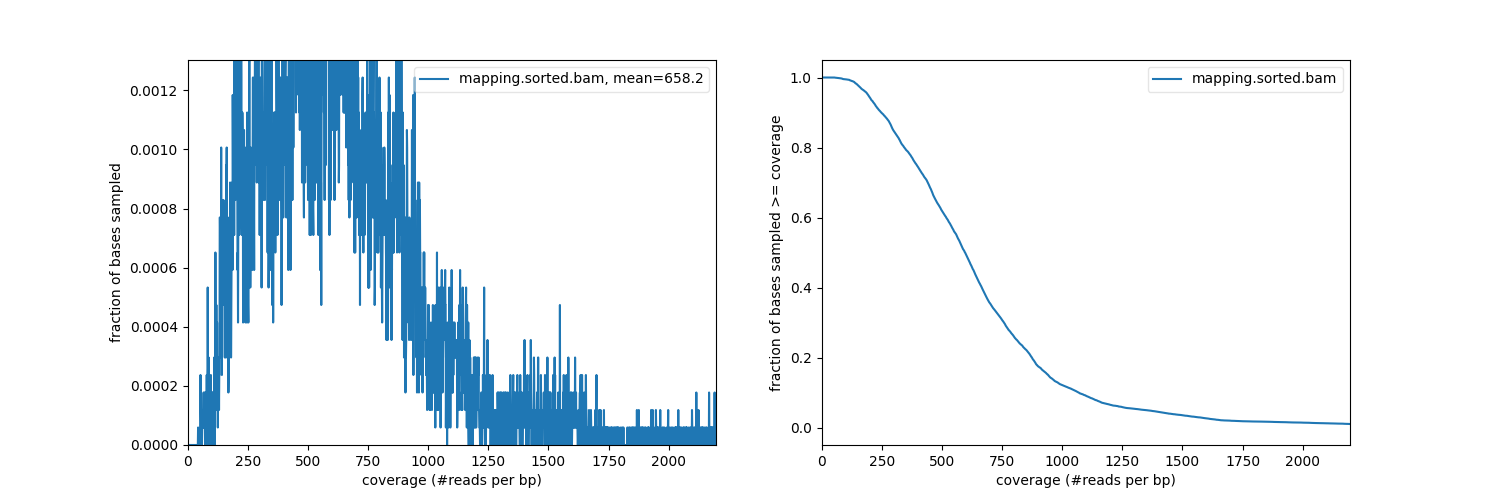
Supplementary Figure S1. Coverage depth plot of the mitochondrial genome of Rhynchodemus sylvaticus, as obtained by plotCoverage.
